# Supplementary material for: Time‑resolved multi-omic analysis of paclitaxel exposure in human iPSC‑derived sensory neurons unveils mechanisms of chemotherapy‑induced peripheral neuropathy
Source: Cell Death Dis. 2026 Feb 10;17(1):211. doi: 10.1038/s41419-026-08445-2 (PMC12921266; doi:10.1038/s41419-026-08445-2)
Supplement: Supplementary file 15 — Dataset 1. [file 41419_2026_8445_MOESM15_ESM.zip › CDD_Open_Data/04_RNA_Seq/Timeline_panel_plots_etc_2023-01-23_1609.zip!/Panel_Plots_etc/Evidence_Plots/post5d/evidence_plot_standard contrasts_msigdb_reactome_5d_PTX_vs_DMSO_ID6_M4281_REACTOME_NEUROTRANSMITTER_RELEASE_CYCLE.pdf]

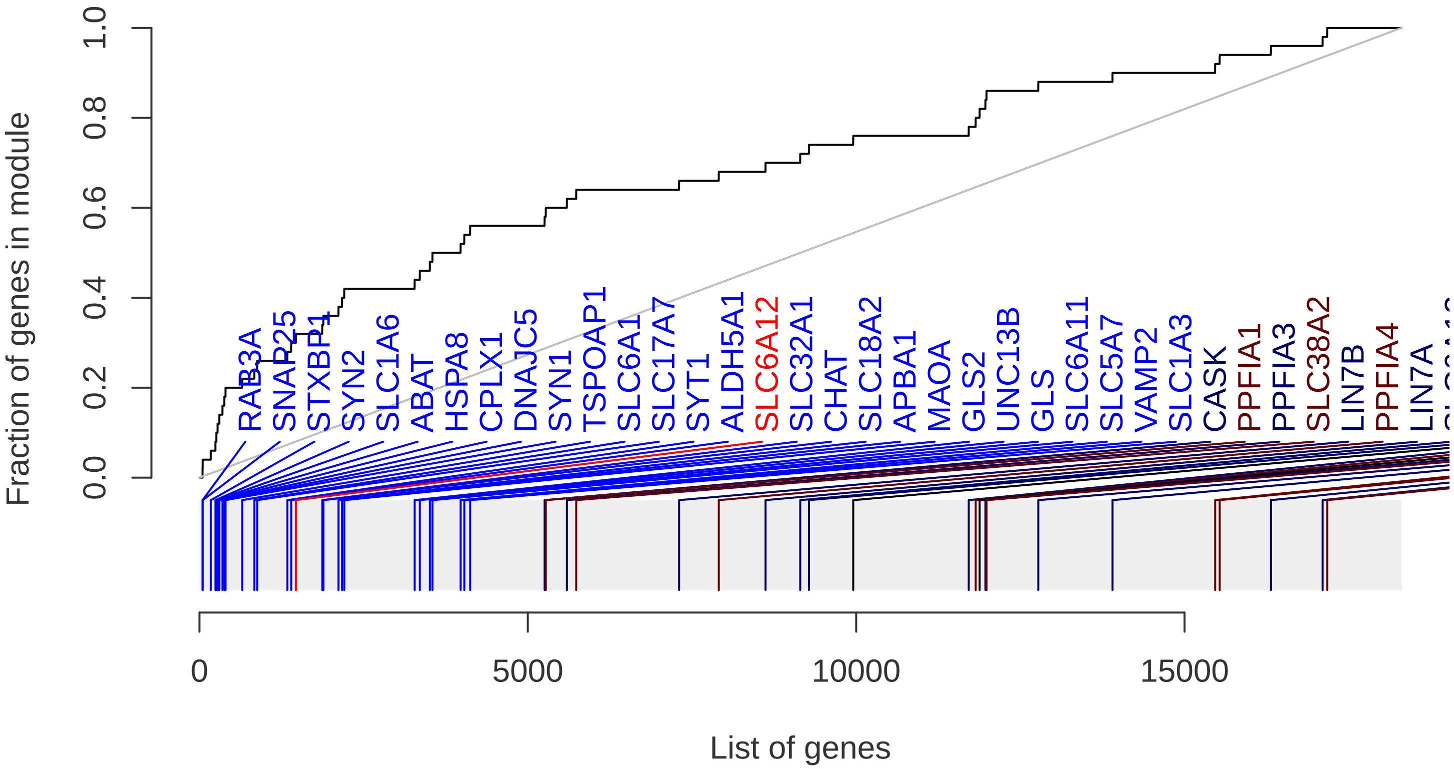
Fraction of genes in module
0.0
0.2
0.4
0.6
0.8
1.0
RAB3A
SNAP25
STXBP1
SYN2
SLC1A6
ABAT
HSPA8
CPLX1
DNAJC5
SYN1
TSPOAP1
SLC6A1
SLC17A7
SYT1
ALDH5A1
SLC6A12
SLC32A1
CHAT
SLC18A2
APBA1
MAOA
GLS2
UNC13B
GLS
SLC6A11
SLC5A7
VAMP2
SLC1A3
CASK
PPFIA1
PPFIA3
SLC38A2
LIN7B
PPFIA4
LIN7A
0
5000
10000
15000
List of genes
